# Adiponectin is a potential catabolic mediator in osteoarthritis cartilage

**DOI:** 10.1186/ar3218

**Published:** 2010-12-31

**Authors:** Eun Ha Kang, Yun Jong Lee, Tae Kyun Kim, Chong Bum Chang, Jin-Haeng Chung, Kichul Shin, Eun Young Lee, Eun Bong Lee, Yeong Wook Song

**Affiliations:** 1 Department of Internal Medicine, Seoul National University Bundang Hospital, 166 Gumi-ro, Bundang-gu, Seongnam-si, Gyeonggi-do, Korea, 463-707; 2 Department of Internal Medicine, Seoul National University College of Medicine, 28 Yeongeon-dong, Jongro-gu, Seoul, Korea, 110-799; 3 Department of Orthopedic Surgery, Seoul National University Bundang Hospital, 166 Gumi-ro, Bundang-gu, Seongnam-si, Gyeonggi-do, Korea, 463-707; 4 Department of Pathology, Seoul National University Bundang Hospital, 166 Gumi-ro, Bundang-gu, Seongnam-si, Gyeonggi-do, Korea, 463-707; 5 Department of Internal Medicine, Seoul National University Hospital, 28 Yeongeon-dong, Jongro-gu, Seoul, Korea, 110-744

## Abstract

**Introduction:**

Adiponectin has been implicated in the pathogenesis of osteoarthritis (OA). We studied the effects of adiponectin on the OA cartilage homeostasis.

**Methods:**

Immunohistochemical analysis was performed to evaluate differential expression of adiponectin receptors (AdipoRs) in nonlesional and lesional areas of OA cartilage. Cartilage and chondrocytes from the knee joints of primary OA patients were cultured in the presence of adiponectin (0~30 μg/ml). The levels of total nitric oxide (NO), matrix metalloproteinase (MMP)-1, -3, and -13, and tissue inhibitor of metalloproteinase (TIMP)-1 were measured in the conditioned media. The levels of inducible NO synthase (iNOS) and MMPs were determined with the quantitative real-time reverse transcription-polymerase chain reaction. The concentrations of collagenase-cleaved type II collagen neoepitope (C1-2C) were determined in the supernatant of adiponectin-stimulated OA cartilage explants. The effects of kinase and NOS inhibitors were evaluated in the adiponectin-stimulated chondrocytes.

**Results:**

The expression levels of both AdipoR1 and AdipoR2 were significantly higher in lesional than in nonlesional areas of OA cartilage. The increased rate of AdipoR1-positive chondrocytes was twice that of AdipoR2-positive chondrocytes when compared between nonlesional and lesional areas. Adiponectin-stimulated OA chondrocytes showed increased total NO and MMP-1, -3, and -13 levels compared with nonstimulated cells. The TIMP-1 level was not affected. The C1-2C levels were increased by adiponectin in OA cartilage explant culture. AMP-activated protein kinase (AMPK) and c-Jun N-terminal kinase (JNK) inhibitors (compound C and SP600125) significantly suppressed adiponectin-induced production of total NO and MMP-1, -3, and -13. Inducible NOS inhibitors enhanced the expression of the adiponectin-induced MMPs.

**Conclusions:**

Adiponectin causes matrix degradation in OA cartilage and increases MMPs and iNOS expression via the AMPK and JNK pathways in human OA chondrocytes. The catabolic effects of adiponectin may be counteracted by NO.

## Introduction

Obesity has long been considered a risk factor for osteoarthritis (OA) [1-4]. It has been reported that obesity increases the incidence of OA, particularly in weight-bearing joints such as knees [4], and weight reduction is correlated with decreased progression of OA. A prevailing hypothesis is that obesity increases mechanical loading across the articular cartilage, which leads to cartilage degeneration [5]. However, obesity also is associated with OA in non-weight-bearing joints such as finger joints, which suggests that metabolic factors contribute to the high prevalence of OA in obese subjects [6].

Adipose tissue is a highly active endocrine organ that secretes many hormones involved in energy metabolism, inflammation, and immune response. Such hormones, collectively termed adipokines, exhibit cytokine-like actions including anti- and pro-inflammatory effects [7]. Adiponectin has been considered one of adipokines implicated in OA pathogenesis, based on the following clinical observations: (a) plasma adiponectin levels were significantly higher in OA patients than in healthy controls [8], and (b) higher plasma adiponectin levels were observed in female patients with erosive hand OA than in those with nonerosive OA [9]. In adddition, adiponectin has been detected in the OA synovial fluids, and its receptors are expressed in the joint tissues [10-13]. However, only few studies examined its biologic roles in OA pathogenesis, and the results have been controversial. Chen *et al*. [12] showed that human cartilage expressed only AdipoR1. However, both AdipoR1 and AdipoR2 were expressed in human cartilage and chondrocytes in the study of Lago *et al. *[13]. In addition, Chen *et al*. [12] reported that adiponectin upregulates tissue inhibitor of metalloproteinase (TIMP)-2 and downregulates IL-1β-induced matrix metalloproteinase (MMP)-13 in OA chondrocytes, whereas Lago *et al*. [13] showed that adiponectin induces nitric oxide synthase (NOS), IL-6, MMP-3, MMP-9, and MCP-1 in murine ATDC5 chondrogenic cell lines. Further to elucidate the effect of adiponectin on OA cartilage homeostasis, we investigated adiponectin-induced catabolic activity in OA chondrocytes and matrix degradation of cartilage explant.

Adiponectin activates intracellular signaling pathway by activation of 5'-AMP-activated protein kinase (AMPK) [14]. It was previously reported that adiponectin stimulates the AMPK-PI3-K pathway in the murine chondrocytic ATDC5 cell line and AMPK/p38/IKKαβ in human synovial fibroblasts [13,15]. However, signaling pathways downstream to AMPK have not been extensively investigated in the human chondrocytes. Therefore, we also studied the intracellular signaling pathways involved in adiponectin-induced MMPs and NO production.

## Materials and methods

### Study subjects

Cartilage was obtained from the knee joints of 12 primary OA patients at the time of knee-replacement surgery (six for immunohistochemical study and six for *in vitro *stimulation experiments). All study subjects had symptomatic OA with Kellgren-Lawrence grade 3 or 4 in their index knees. They were all women with a mean age of 71.4 years (range, 59 to 80 years), and their mean body mass index (BMI) was 26.1 kg/m^2 ^(range, 21.4 to 30.1 kg/m^2^). This study was approved by the Institutional Review Board of Seoul National University Bundang Hospital (IRB No. B-0607/035-018), and written informed consent was obtained from study participants.

### Assessment of AdipoR1 and AdiopoR2 expression by immunohistochemistry

The postsurgical femoral cartilage samples obtained from six patients (mean age, 70.0 years; mean BMI, 26.8 kg/m^2^) were fixed in 4% buffered paraformaldehyde for 2 days and decalcified with buffered EDTA (20% EDTA, pH 7.4). After dehydration and embedding in paraffin, sections were cut at a thickness of 4 μm, deparaffinized in xylene, and rehydrated in graded ethanol. Serial sections from each case were stained with hematoxylin and eosin and rabbit antibodies against human AdipoR1 (Phoenix Pharmaceuticals, St Joseph, MO, USA; catalog no. H-001-44) and AdiopR2 (catalog no. H-001-23). The succeeding steps were performed automatically at 37°C by using the Benchmark XT Slide Staining System Specifications (Ventana Medical Systems, Tucson, AZ, USA). Antigen retrieval was performed by immersing slides in citrate buffer (pH 6.0) for 15 minutes, and endogenous peroxidases were blocked with 1% H_2_O_2 _for 4 minutes. The sections were incubated with anti-human adiponectin receptors at the dilution of 1:100 for 60 minutes at room temperature. To visualize the immunostaining, the Ultravision LP kit (Lab Vision, Fremont, CA, USA) was used. The slides were stained by using a diaminobenzidine (DAB) detection kit and counterstained with hematoxylin. Specimens were evaluated under light microscopy by an expert pathologist (J-H.C.) and scored based on a semiquantitative approach of percentage of positive chondrocytes (0 to 100%) and staining intensity (0, negative; 1, weak; 2, moderate; 3, strong) in the lesional and nonlesional areas of each cartilage sample. The number of stained cells (staining intensity score, ≥1) and total cells were counted in at least three randomly selected high-power fields (150 cells or more) for each area of cartilage samples.

### Primary culture of OA chondrocytes

The cartilage portions with less than 50% of thickness loss were harvested from postsurgical cartilage samples of another six patients (mean age, 72.6 years; mean BMI, 26.1 kg/m^2^), and chondrocytes were released by enzymatic digestion with 0.2% pronase (Sigma-Aldrich, St. Louis, MO, USA) and 0.3% clostridial collagenase (Worthington, Freehold, NJ, USA). Isolated chondrocytes were plated in poly-2 hydroxyethyl methacrylate (HEMA; Sigma-Aldrich)-coated 60-mm-diameter dishes (1 × 10^6 ^cells/dish) or 24-well plates (2.5 × 10^5 ^cells/well) and cultured in Dulbecco's Modified Eagle Medium (DMEM) containing 10% fetal bovine serum (FBS), 100 IU/ml penicillin, and 100 μg/ml streptomycin at 37°C in a humidified 5% CO_2 _atmosphere. The culture medium was changed every 2 to 3 days in suspension culture, and chondrocytes were stimulated 5 to 6 days after isolation. Nonadherent culture in HEMA-coated dishes has been described as a means of maintaining the chondrocyte-specific phenotype for up to 3 months [16]. To prepare a 10 × stock solution, poly-HEMA was dissolved at 120 mg/ml in 95% ethanol, and the solution was incubated overnight at 37°C. After removal of undissolved materials, the stock solution was diluted with 95% ethanol to a final concentration of 12 mg/ml. Culture dishes or plates were coated with 0.1 ml/cm^2 ^of the diluted poly-HEMA solution and then air-dried uncovered in a sterile environment for 2 days.

### Cell treatments

OA chondrocytes were stimulated with the full-length adiponectin at 0, 1, 10, or 30 μg/ml for 24 hours (AdipoGen, Seoul, South Korea) in FBS-free DMEM. The full-length adiponectin used in our study was a lyophilized form of the FLAG-tagged recombinant human adiponectin expressed by HEK 293 cells. When indicated, NOS inhibitors were added in the presence of adiponectin; 2 m*M *L-*N*^*G*^-monomethyl arginine citrate (L-NMMA; Calbiochem, San Diego, CA, USA), a nonselective NOS inhibitor, and 50 μ*M *of L-*N*^*6*^-(1-iminoethyl)-lysine (L-NIL; BioMol International, Plymouth Meeting, MA, USA), a selective iNOS inhibitor.

To ascertain the adiponectin-related signaling pathways, OA chondrocytes were stimulated with adiponectin in the presence of a kinase inhibitor; 10 μ*M *SB202190 for p38 MAP kinase (Alexis Biochemicals, Farmingdale, MI, USA), 20 μ*M *SP600125 for c-Jun N-terminal kinase (JNK; BioMol International), 50 μ*M *U0126 for extracellular-regulated kinase (ERK; Promega, Madison, WI, USA), 20 μ*M *compound C for AMP-activated protein kinase (AMPK; Calbiochem), 50 μ*M *LY294002 for Akt (BioSource International, Camarillo, CA, USA), and 100 μg/ml SN50 for nuclear factor kappa B (NF-κB; Alexis Biochemicals). No significant cytotoxicity was found for OA chondrocytes by the kinases or NOS inhibitors up to 24 hours of exposure (data not shown).

### Measurement of NO and MMPs/TIMP-1 levels in culture media

The levels of total NO were measured by using a modified Griess reaction (Promega). The concentrations of MMP-1, -3, and -13 and TIMP-1 in the conditioned media were analyzed by using commercial enzyme-linked immunosorbent assay kits (ELISA; catalog no. DMP100 for MMP-1, DMP300 for MMP-3, DM1300 for MMP-13, and DTM100 for TIMP-1, R&D Systems, Minneapolis, MN, USA), which measured the pro-MMP forms of MMP-1 and MMP-13 and the total forms for MMP-3.

### Western blotting

iNOS expression in adiponectin-stimulated OA chondrocytes was analyzed by immunoblotting by using anti-iNOS (BD Biosciences, San Jose, CA, USA) and goat anti-rabbit antibody (Zymed, San Francisco, CA, USA). Adiponectin-stimulated activation of AMPK and JNK was evaluated by using anti-phospho-AMPK and phospho-JNK antibodies (Cell Signaling, Boston, MA, USA).

### Reverse transcription polymerase chain reaction (RT-PCR)

RNA expression levels of iNOS and MMPs were semiquantitatively determined by using the RT-PCR with specific primer pairs; 5'-TCATCTTCGCCACCAAGCAGG-3' and 5'-AGCATTCCACACCCGGAAGTC-3' for iNOS (GenBank Accession Number NM_000625), 5'-CCTTCTACCCGGAAGTTGAG-3' and 5'-TCCGTGTAGCACATTCTGTC-3' for MMP-1 (NM_002421), 5'-GAAAGTCTGGGAAGAGGTGAC-3' and 5'-AACCGAGTCAGGTCTGTGAG-3' MMP-3 (NM_002422), and 5'-GAATTAAGGAGCATGGCGAC-3' and 5'-TAAGGAGTGGCCGAACTCAT-3' for MMP-13 (NM_002427). β-actin was used as the internal RT-PCR control by using forward primer 5'-ACACTGTGCCCATCTACGAG-3' and reverse primer 5'-TACAGGTCTTTGCGGATGTC-3' (NM_001101).

Quantitative real-time RT-PCR was performed by using the ABI-7500 real-time PCR machine (Applied Biosystems, Foster City, CA, USA). The specific Taqman primers and probes were purchased from Applied Biosystems; iNOS (assay ID Hs00167248_m1), MMP-1 (Hs00233958_m1), MMP-3 (Hs00968308_m1), MMP-13 (Hs00233992_m1), and glyceraldehyde-3-phosphate dehydrogenase (GAPDH; Hs99999905). The number-fold difference in the expression of target mRNA was calculated by a comparative Ct method (2^-ΔΔCt^), normalized to GAPDH.

### Measurement of collagenase-cleaved type II collagen neoepitope

To assess cartilage matrix degradation, the harvested OA cartilage tissue was cut into cubes of approximately 1 × 1 × 1 mm in size by using surgical blades. Cartilage pieces weighing a total of approximately 200 mg were placed into each well of a 24-well tissue plate with 1 ml/well of DMEM supplemented with 10% FBS. After 2 to 3 days, the cartilage explants were stimulated with FBS-free DMEM including adiponectin (30 μg/ml) or interleukin (IL)-1β (5 ng/ml) for 8 days. During the treatment, the conditioned medium was harvested and replaced every 4 days. The concentrations of collagenase-cleaved type II collagen product (C1-2C or COL2 ¾ C short) were measured in the harvested media by using a competitive immunoassay kit (catalog no. 60-1002-001; IBEX Pharmaceuticals, Montreal, QC, Canada) on days 4 and 8 after adiponectin treatment. In brief, 50 μl/well of sample and 50 μl/well of diluted anti-C1-2C antibody were preincubated in a polypropylene mixing plate for 30 minutes at room temperature. Eighty microliters per well of the mixture was transferred to another ELISA plate. After incubation for 1 hour and washing, 100 μl/well of goat anti-rabbit horseradish peroxidase (HRP) conjugate was added and incubated for 30 minutes. After repeated washing, the plate was incubated for 30 minutes and then treated with tetramethylbenzidine for another 30 minutes. The reaction was stopped by using 100 μl/well of 0.2 *M *sulfuric acid, and absorbance was measured at 450 nm.

### Statistical analysis

Total NO, MMPs, TIMP-1, and C1-2C levels in the conditioned media were measured in duplicate. Quantitative real-time RT-PCR was performed in triplicate. To compensate for interindividual variations, adipokine-induced NO and MMPs/TIMP-1 levels are presented as ratios versus nonstimulated levels. Continuous values are presented as mean ± SEM. Statistical significance was determined with the Mann-Whitney *U *test or Wilcoxon matched-pairs signed-rank test using SPSS for Windows version 11.0 (SPSS, Chicago, IL, USA), and *P *values of < 0.05 were considered significant.

## Results

### Adiponectin receptors expression in OA cartilage

**Figure 1:**
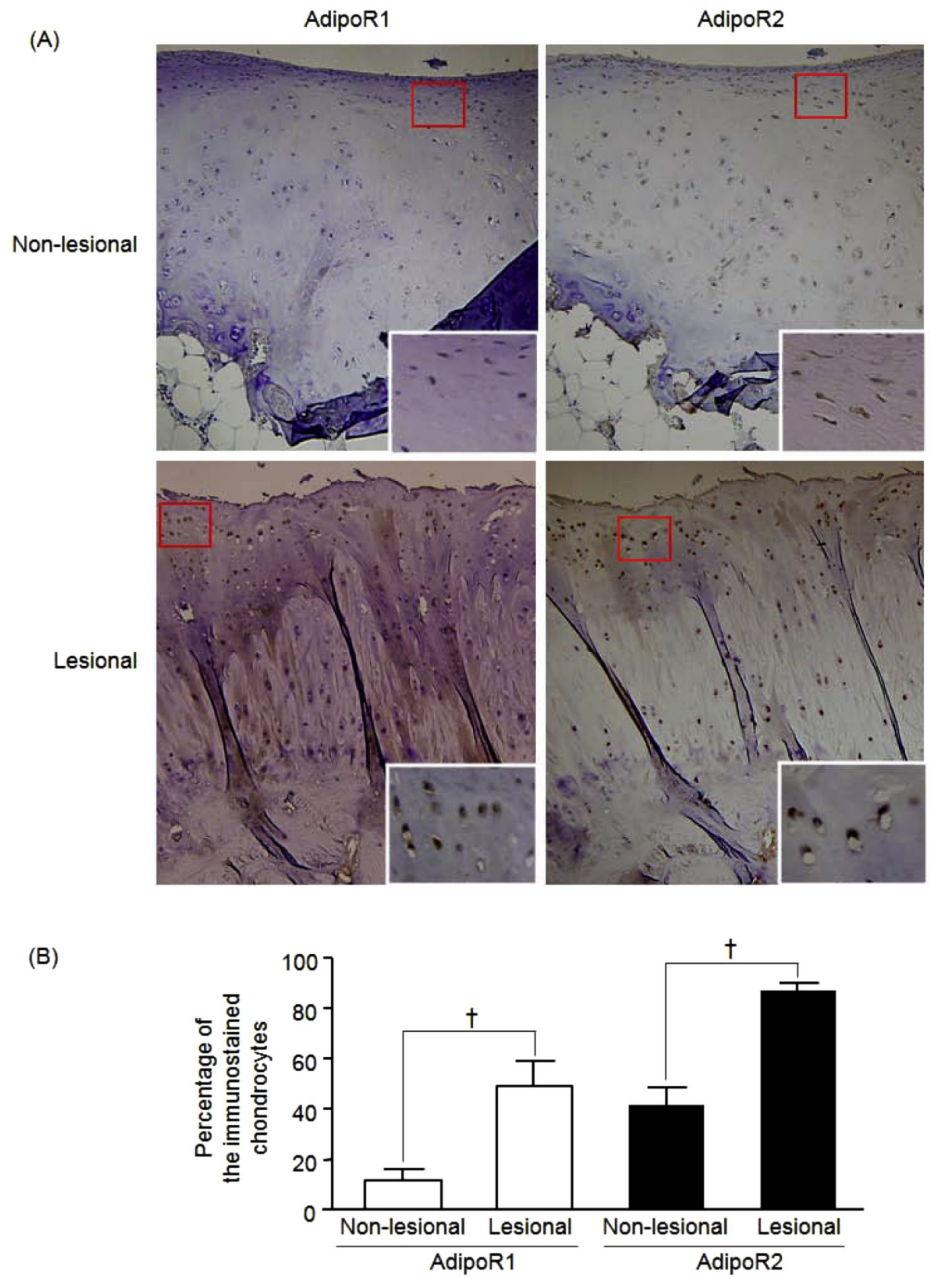
**Immunohistochemical staining for adiponectin receptor type 1 and type 2**. Representative cartilage slides showed the expression of AdipoR1 or AdipoR2 (**aA**, original magnification × 100). A nonlesional cartilage area revealed weakly AdipoR2-positive chondrocytes, but not AdipoR1-positive chondrocytes. In a lesional cartilage area, AdipoR1 was stained mainly in the superficial layer (weak to strong staining intensity), whereas AdipoR2 was stained in both superficial and deep layers of the lesional cartilage (strong staining intensity). The insert is a magnified view (×400) of the area indicated by a red box. When compared between nonlesional and lesional areas, the expression levels of these two receptors significantly increased in lesional areas (**B**, †*P *< 0.05 by Wilcoxon matched-pairs signed-rank test), and the increased rate of AdipoR1 (4.2 times) was as twice as high as that of AdipoR2 (2.1 times).

Immunohistochemical study demonstrated that all OA cartilage samples expressed both AdipoR1 and AdipoR2; AdipoR2 was expressed through all layers, whereas AdipoR1 was expressed mainly in the superficial layer of OA cartilage (Figure 1a). Both AdipoR1 (49.0 ± 9.9% versus 11.7 ± 4.6%; *P *< 0.05 by Wilcoxon matched-pairs signed-rank test) and AdipoR2 (87.2 ± 2.7% versus 41.7 ± 6.9%; *P *< 0.05) were significantly more expressed in the lesional cartilage area than in the nonlesional area. When the expression levels of AdipoR1 and AdipoR2 were compared, the AdipoR2 was more strongly stained than AdipoR1 in both nonlesional (staining intensity score ranges from 1~2 versus 0~1) and lesional area (staining intensity score ranges, 3 versus 1~3). Additionally, the percentage of AdipoR2-positive chondrocytes was significantly higher than that of AdipoR1-positive chondrocytes in both nonlesional and lesional areas (each *P *< 0.05). However, the counts of AdipoR1-stained chondrocytes were increased at a higher rate than those of AdipoR2-stained chondrocytes (4.2 versus 2.1 times; Figure 1b). The percentages of AdipoR1- or AdipoR2-positive chondrocytes were not shown to be correlated with either age or BMI.

### Effects of adipokines on total NO production and iNOS expression

**Figure 2:**
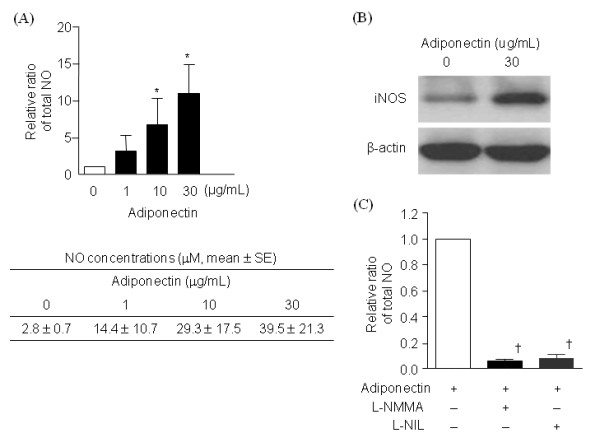
**Total NO production and iNOS expression in adiponectin-stimulated osteoarthritis (OA) chondrocytes**. Adiponectin significantly increased total NO production (*n *= 6, duplicate, *P *< 0.01) **(A)**. Western blotting showed upregulation of iNOS expression in OA chondrocytes stimulated with 30 μg/ml adiponectin **(B)**. Both nonselective (L-NMMA, 2 m*M*) and selective NOS inhibitors (L-NIL, 50 μ*M*) significantly prevented total NO secretion (*n *= 4, duplicate, *P *< 0.05 for both) **(c)**. The histogram with error bars represents the mean and the standard error of the mean. *P < 0.01 by Mann-Whitney test; †*P *< 0.05 by Mann-Whitney test.

Adiponectin-stimulated OA chondrocytes (the number of samples, *n *= 6) significantly increased total NO production in a dose-dependent manner (Figure 2a). Adiponectin was also found to upregulate iNOS levels (Figure 2b). Furthermore, adiponectin-induced NO production was significantly inhibited by NOS inhibitors, L-NMMA and L-NIL (*n *= 4) (Figure 2c).

### Effects of adipokines on MMP-1, MMP-3, MMP-13 and TIMP-1 secretion

**Figure 3:**
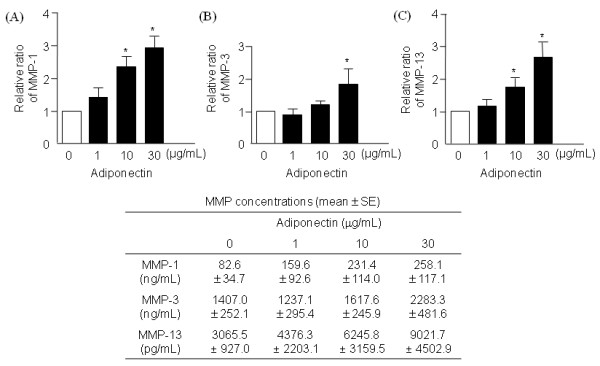
**Effects of adiponectin on the expression of matrix metalloproteinase (MMP)-1, MMP-3, and MMP-13 in osteoarthritis (OA) chondrocytes**. In the conditioned media, adiponectin significantly increased the levels of MMP-1 **(A) **and MMP-3 **(B)**, and MMP-13 **(c) **(*n *= 6, duplicate, *P *< 0.01 for all). The histogram with error bars represents the mean and the standard error of the mean. *P < 0.01 by Mann-Whitney test; †*P *< 0.05 by Mann-Whitney test.

**Figure 4:**
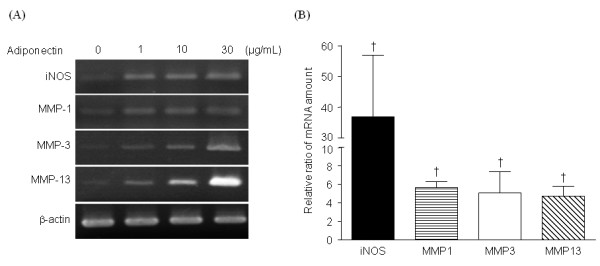
**Reverse transcription polymerase chain reaction (RT-PCR) for iNOS and MMPs**. Representative semiquantitative RT-PCR result for iNOS and matrix metalloproteinases (MMPs) **(A)**. Real-time RT-PCR demonstrated that adiponectin significantly increased the transcripts of iNOS and all three MMPs in the osteoarthritis (OA) chondrocytes (*n *= 4, triplicate, *P *< 0.05) at 30 μg/ml **(B)**. The histogram with error bars represents the mean and the standard error of the mean. †*P *< 0.05 by Mann-Whitney test.

Adiponectin increased the concentrations of MMP-1 (Figure 3a), MMP-3 (Figure 3b), and MMP-13 (Figure 3c) in the supernatants in a dose-dependent manner (*n *= 6). However, TIMP-1 levels were not changed. Consistent with ELISA results, quantitative RT-PCR showed that MMP-1, -3, and -13 mRNA levels were upregulated by 30 μg/ml of adiponectin (*n *= 4; Figure 4).

### Effects of adipokines on the degradation of OA cartilage matrix

**Figure 5:**
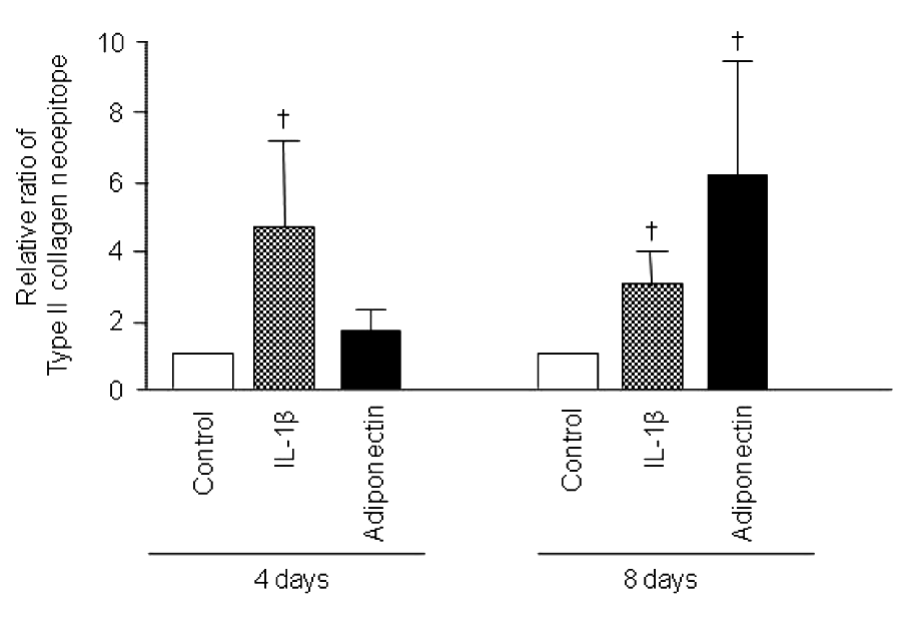
**Effect of adiponectin on the degradation of osteoarthritis (OA) cartilage matrix**. IL-1β (5 ng/ml) significantly increased the concentrations of collagenase-cleaved type II collagen neoepitope (C1-2C) in conditioned media collected on days 4 and 8 (*n *= 4, duplicate, *P *< 0.05 for both). Adiponectin (30 μg/ml) also showed increased the C1-2C levels in the media on day 8 (*P *< 0.05). The histogram with error bars represents the mean and the standard error of the mean. †*P *< 0.05 by Mann-Whitney test.

The effect of the adiponectin on matrix degradation in OA cartilage explants (*n *= 4) was evaluated *ex vivo *(Figure 5). IL-1β served as a positive control. On days 4 and 8, the levels of C1-2C were significantly increased in the supernatants of cartilage explants cultures by 5 ng/ml of IL-1β. In the meantime, C1-2C concentrations were significantly elevated on day 8 with 30 μg/ml adiponectin.

### Effect of protein kinase inhibitors on adiponectin-induced production of MMPs and NO

**Figure 6:**
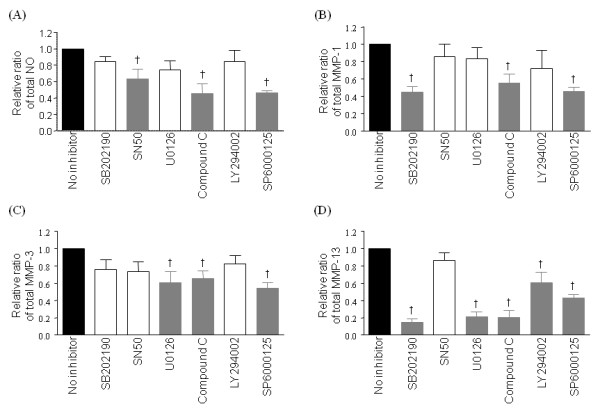
**Effects of kinase inhibitors on the adiponectin-induced production of total NO (A), matrix metalloproteinase (MMP)-1 (B), MMP-3 (C), and MMP-13 (D) in osteoarthritis (OA) chondrocytes**. AMPK and JNK inhibitors (20 μ*M *compound C and SP6000125, respectively) significantly inhibited the production of total NO and all three MMPs (*P *< 0.05). NF-κB (inhibited by 100 μg/ml SN50) was involved in NO production, p38 MAP kinase (inhibited by 10 μ*M *SB202190) in MMP-1 and -13, ERK (inhibited by 50 μ*M *U0126) in MMP-3 and -13, and Akt kinase (inhibited by 50 μ*M *LY294002) in MMP-13 expressions. Experiments were performed on four samples in duplicates. The histogram with error bars represents the mean and the standard error of the mean. The gray bars indicate significantly decreased total NO or MMP levels by Mann-Whitney *U *test (†*P *< 0.05).

**Figure 7:**
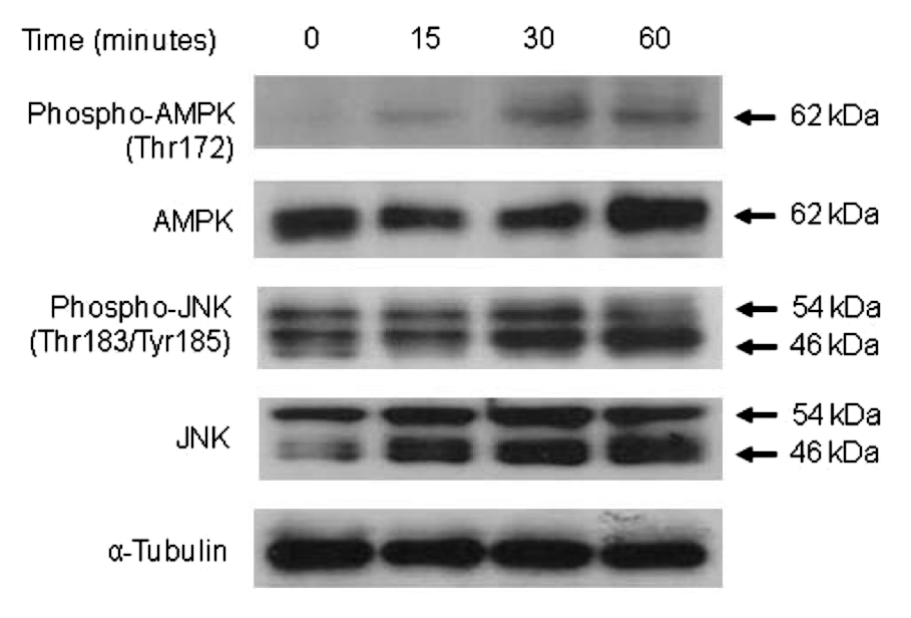
**Adiponectin-induced AMPK and JNK phosphorylation**. When osteoarthritis (OA) chondrocytes were stimulated with 30 μg/ml adiponectin, AMPK phosphorylation was increased after 15 minutes and peaked at 30 minutes. The levels of phospho-JNK were increased 30 minutes after adiponectin stimulation.

Because adiponectin was a potential player in cartilage degradation *in vitro *and *ex vivo*, we assessed signaling pathways involved in adipokine-induced upregulation of NO and MMPs. After plating OA chondrocytes (*n *= 4) in wells coated with poly-HEMA, protein kinases were added to the media 1 hour before adiponectin treatment (30 μg/ml), and cells were incubated for 24 hours. Adiponectin-induced total NO production was significantly suppressed by inhibitors of NF-κB, AMPK, and JNK (Figure 6a). In addition, MMP-1 secretion was inhibited by p38, AMPK, or JNK inhibitors (Figure 6b), MMP-3 by ERK, AMPK, and JNK inhibitors (Figure 6c), and MMP-13 by all but NF-κB inhibitor (Figure 6d). Especially AMPK and JNK inhibitors significantly suppressed production of total NO and all three MMPs by 40% or more, suggesting that AMPK-JNK axis is the major pathway involved in adiponectin-induced biologic actions. When examined with immunoblotting, increased phospho-AMPK and phospho-JNK levels were observed in adiponectin-stimulated OA chondrocytes (Figure 7).

### Effect of NOS inhibitors on adiponectin-induced production of MMPs

**Figure 8:**
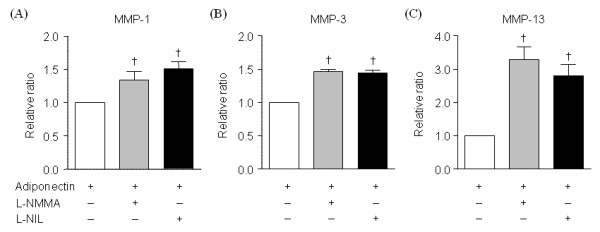
**Effects of iNOS inhibitors on the adiponectin-induced production of matrix metalloproteinases MMPs in osteoarthritis (OA) chondrocytes**. A nonselective NOS inhibitor (L-NMMA, 2 m*M*) and a specific iNOS inhibitor (L-NIL, 50 μ*M*) significantly enhanced MMP-1 **(A)**, MMP-3 **(B)**, and MMP-13 **(C) **expressions induced by adiponectin (30 μg/ml). Experiments were performed on four samples in duplicates. The histogram with error bars represents the mean and the standard error of the mean. †*P *< 0.05 by Mann-Whitney test.

Because adiponectin markedly enhanced NO production in OA chondrocytes in the present study and because NO has been previously suggested to affect the expression of MMPs [17,18], the effects of NOS inhibitors on adiponectin-induced MMPs production were evaluated by using a nonselective NOS inhibitor, L-NMMA, and a selective iNOS inhibitor, L-NIL. Interestingly, when the NOS inhibitors were added to chondrocytes 24 hours before adiponectin stimulation, both inhibitors significantly augmented adiponectin-induced secretion of the three MMPs (*n *= 4) (Figure 8). Especially the levels of MMP-13 were increased by an average of 3.3-fold with L-NMMA and by an average of 2.8-fold with L-NIL.

## Discussion

The present study demonstrates that adiponectin increased NO and three MMPs production in human OA chondrocytes mainly via the AMPK-JNK pathway *in vitro *and that adiponectin-induced NO and MMPs lead to accelerated degradation of OA cartilage matrix *ex vivo*.

Our *in vitro *findings indicate that adiponectin is a potential catabolic mediator in OA. This is in line with the previous findings that adiponectin induces iNOS, MMP-3, MMP-9, and MCP-1 in murine chondrocytes [13]. More important, increased cartilage degradation products after adiponectin treatment further supports that *in vitro *catabolic activity induced by adiponectin is relevant to cause cartilage degradation. Our result is in parallel with the result of a recent study indicating that the synovial fluid levels of adiponectin are correlated with aggrecan degradation markers in patients with knee OA [19]. However, Chen *et al*. [12] reported that adiponectin did not alter the expression levels of MMP-3 and MMP-13 mRNA. The contrasting results regarding the effect of adiponectin might be due to experimental conditions. Chen *et al*. used chondrocytes from the OA knees with diverse severities and evaluated the effects in monolayered cells at passages 3 to 7 [12], whereas we isolated chondrocytes from the OA knees with Kellgren-Lawrence grade 3 or 4 and grew them in suspension at passage 0. Because OA chondrocyte behavior and phenotypes can be affected by the surrounding matrix state, culture methods, and passage numbers [20], this might have contributed to the difference of adiponectin-induced responses in each study.

Another possibility is a different composition of adiponectin isoforms due to a different biologic source from which adiponectin is produced. Native adiponectin has a multimeric structure and circulates in blood as trimers, hexamers, and high-molecular-weight (HMW) complexes [14]. Biologic effects mediated by adiponectin have been considered to be isoform dependent. HMW adiponectin has pro-inflammatory effects [21,22], whereas the low-molecular-weight (LMW) isoform has antiinflammatory functions in human leukocytes and monocytic cells [23,24]. We used HEK293 cell-derived full-length adiponectin, the most abundant isoforms of which are hexamers and HMW forms, followed by trimers [25]. This composition is similar to that of human OA synovial fluid in which hexamers and HMW forms are the most abundant isoforms [12]. Conversely, full-length adiponectin derived from *Escherichia coli *lacks HMW forms [25]. Morevoer, adiponectin of the same isoform could display a different potency to induce a biologic response depending on whether it is *E. coli *derived or mammalian cell derived [25,26]; adiponectin produced in mammalian cells seems to be functionally more potent than bacterially produced adiponectin because the HMW form is a predominantly active form. Because it is believed that *E. coli*-derived adiponectin was used in the previous studies [12,13], pro-inflammatory effects of adiponectin might not have been fully developed in those studies.

Biologic effects of adiponectin are mediated mainly through two receptors, AdipoR1 and AdipoR2, and these two receptors are believed to activate different signaling pathways; AdipoR1 activates the AMPK pathway, whereas AdipoR2 is linked more closely with the peroxisome proliferator-activated receptor α (PPAR-α) pathway in the liver [27]. Chen *et al*. [12] showed that human cartilage expressed only AdipoR1. However, our study showed that both AdipoR1 and AdipoR2 are expressed in human cartilage tissue, consistent with the results of Lago *et al. *[13]. A heterogeneous distribution of AdipoR1 and AdipoR2 on chondrocytes might be a factor that explains the difference between our results and those of the others. In our study, the expression of AdipoR2 was higher in terms of the immunostaining intensity as well as the percentage of stained cells, but the increase rate of AdipoR1 was as twice as high as that of AdipoR2, when nonlesional and lesional cartilage areas were compared. This finding suggests that the change of AdipoR1 expression might better reflect the cartilage catabolic state than that of AdipoR2, and that the AdipoR1-AMPK pathway could be associated with cartilage catabolism.

It has been well established that adiponectin activates AMPK [14]. Lago *et al*. [13] reported that the AMPK/Akt signaling pathway is involved in iNOS and MMP-3 induction by adiponectin in the murine chondrocyte ATDC5 cell line. In addition, adiponectin activated the AMPK/p38/NF-κB axis in human synovial fibroblasts to induce IL-6 production [15]. Conversely, in our study, AMPK/JNK pathways are the major signaling pathway involved in adiponectin-mediated induction of iNOS and MMPs in human OA chondrocytes, whereas the AMPK/Akt or AMPK/p38 pathway is partially involved in MMP-13 or MMP-3 induction, respectively. The JNK pathway is one of the signaling intermediates activated by adiponectin [28,29], and adiponectin-induced JNK activation has been shown to follow AMPK activation [30,31]. Furthermore, JNK is involved in MMPs and iNOS expression in human articular chondrocytes [32-36]. Therefore, we expect that adiponectin induces iNOS and MMP expression via JNK downstream to AMPK in human chondrocytes and that the AMPK/JNK axis is a major signaling system responsible for the adiponectin-induced degradation of cartilage matrix.

Because NO can upregulate the expression or activity of MMPs [17,18], we determined whether NO mediates adiponectin-induced synthesis of MMPs. Unexpectedly, the expression of MMPs was further increased by adiponectin after pretreatment with a nonspecific NOS and a specific iNOS inhibitor. This finding is consistent with the previous observation by Hattori *et al*. [37] in which adiponectin-induced NF-κB activation was further enhanced by a nonspecific NO inhibitor, L-NMMA, in human umbilical vein endothelial cells. Interestingly, LY294002, a PI3-K/Akt kinase inhibitor, significantly suppressed NO production, whereas it caused a higher MMP-3 production in adiponectin-treated ATDC5 cells in the study of Lago *et al. *[13]. In this context, we are tempted to speculate that NO serves as a negative-feedback regulator of adiponectin action in cartilage, and that this negative feedback may lead to the delayed effects of adiponectin on the OA cartilage catabolism when compared with those of IL-1β in our study. The role of NO as a catabolic mediator has been controversial. The protective effect of NO on cartilage degradation has been shown by several studies [38-40], in which the treatment with NOS inhibitors accelerated the proteoglycan breakdown by increasing MMP levels in culture media [38]. Thus, the exact role of NO in cartilage homeostasis seems to be complex. Further studies on the effect of NO on AMPK or JNK activation in chondrocytes will elucidate the mechanisms by which NO influences adiponectin-induced MMP production.

We used the highest dosage (30 μg/ml) of adiponectin with maximal biologic activity to investigate the full catabolic potential of adiponectin. Because adiponectin concentrations in OA synovial fluid are typically lower (1 to 5 μg/ml) than the doses used in our study [11], a possibility exists that the catabolic effect of adiponectin is overemphasized in our study. However, the human OA joint tissues including cartilage were reported to release adiponectin in *ex vivo *culture study [11], and ATDC5 cells have been shown to express adiponectin themselves in an autocrine manner [41]. Therefore, the actual concentrations of adiponectin might be higher in the microenvironment surrounding chondrocytes than those measured in OA synovial fluid.

## Conclusions

The present study suggests that adiponectin induces MMPs and iNOS expression via the AMPK/JNK pathway, and it may play a potential role in OA cartilage catabolism.

## Abbreviations

AdipoR: adiponectin receptor; AMPK: AMP-activated protein kinase; C1-2C: collagenase-cleaved type II collagen neoepitope; DMEM: Dulbecco's Modified Eagle Medium; FBS: fetal bovine serum; ELISA: enzyme-linked immunosorbent assay kits; ERK: extracellular-regulated kinase; HEMA: hydroxyethyl methacrylate; iNOS: inducible nitric oxide synthase; JNK: c-Jun N-terminal kinase; L-NIL: L-*N*^6^-(1-iminoethyl)-lysine; L-NMMA: L-*N*^G^-monomethyl arginine citrate; MMP: matrix metalloproteinase; NF-κB: nuclear factor kappa B; NO: nitric oxide; NOS: nitric oxide synthase; OA: osteoarthritis; RT-PCR: reverse transcription polymerase chain reaction; TIMP: tissue inhibitor of metalloproteinase.

## Competing interests

The authors declare that they have no competing interests.

## Authors' contributions

EHK was involved in the acquisition and interpretation of data and prepared the initial draft of the manuscript. YJL conceived and designed the study, participated in the analysis and interpretation of data, and revised the manuscript. TKK and CJB participated in the design of the study, collected the OA cartilage samples, and were involved in the critical revision of the manuscript. JHC was involved in the acquisition and interpretation of immunohistochemical data and in the revision of the manuscript. KS, EYL, EBL, and YWS participated in the design of the study and were involved in the analysis and interpretation of the data and in the critical revision of the manuscript. All authors read and approved the final manuscript.
